# Alpha-1 antitrypsin deficiency: outstanding questions and future directions

**DOI:** 10.1186/s13023-018-0856-9

**Published:** 2018-07-11

**Authors:** María Torres-Durán, José Luis Lopez-Campos, Miriam Barrecheguren, Marc Miravitlles, Beatriz Martinez-Delgado, Silvia Castillo, Amparo Escribano, Adolfo Baloira, María Mercedes Navarro-Garcia, Daniel Pellicer, Lucía Bañuls, María Magallón, Francisco Casas, Francisco Dasí

**Affiliations:** 1 Pulmonary Department, Hospital Álvaro Cunqueiro EOXI, Vigo, Spain; 2 NeumoVigo I+i Research Group, IIS Galicia Sur, Vigo, Spain; 3 Unidad Médico-Quirúrgica de Enfermedades Respiratorias, Instituto de Biomedicina de Sevilla (IBiS), Hospital Universitario Virgen del Rocio, Universidad de Sevilla, Sevilla, Spain; 4 CIBER de Enfermedades Respiratorias (CIBERES), Madrid, Spain; 5 Pneumology Department, Hospital Universitari Vall d’Hebron, Barcelona, Spain; 6 Molecular Genetics Unit, Instituto de Investigación de Enfermedades Raras (IIER), Instituto de Salud Carlos III (ISCIII), Madrid, Spain; 7 Fundación Investigación Hospital Clínico Valencia, Instituto de Investigación Sanitaria INCLIVA, c/Menéndez y Pelayo, 4, 46010 Valencia, Spain; 8 School of Medicine, Department of Paediatrics, Obstetrics and Gynaecology, University of Valencia, Valencia, Spain; 9 Pneumology Department, Complejo Hospitalario Universitario de Pontevedra, Pontevedra, Spain; 10 School of Medicine, Department of Physiology, Research group on Rare Respiratory Diseases (ERR), University of Valencia, Valencia, Spain; 11 Pneumology Department, Hospital Universitario San Cecilio, Granada, Spain

**Keywords:** Rare respiratory diseases, Alpha-1 antitrypsin, Alpha-1 antitrypsin deficiency, SERPINA1, Augmentation therapy, COPD, Cirrhosis, Panniculitis, Vasculitis

## Abstract

**Background:**

Alpha-1 antitrypsin deficiency (AATD) is a rare hereditary condition that leads to decreased circulating alpha-1 antitrypsin (AAT) levels, significantly increasing the risk of serious lung and/or liver disease in children and adults, in which some aspects remain unresolved.

**Methods:**

In this review, we summarise and update current knowledge on alpha-1 antitrypsin deficiency in order to identify and discuss areas of controversy and formulate questions that need further research.

**Results:**

1) AATD is a highly underdiagnosed condition. Over 120,000 European individuals are estimated to have severe AATD and more than 90% of them are underdiagnosed.

**Conclusions:**

2) Several clinical and etiological aspects of the disease are yet to be resolved. New strategies for early detection and biomarkers for patient outcome prediction are needed to reduce morbidity and mortality in these patients; 3) Augmentation therapy is the only specific approved therapy that has shown clinical efficacy in delaying the progression of emphysema. Regrettably, some countries reject registration and reimbursement for this treatment because of the lack of larger randomised, placebo-controlled trials. 4) Alternative strategies are currently being investigated, including the use of gene therapy or induced pluripotent stem cells, and non-augmentation strategies to prevent AAT polymerisation inside hepatocytes.

## Background

**Fig. 1:**
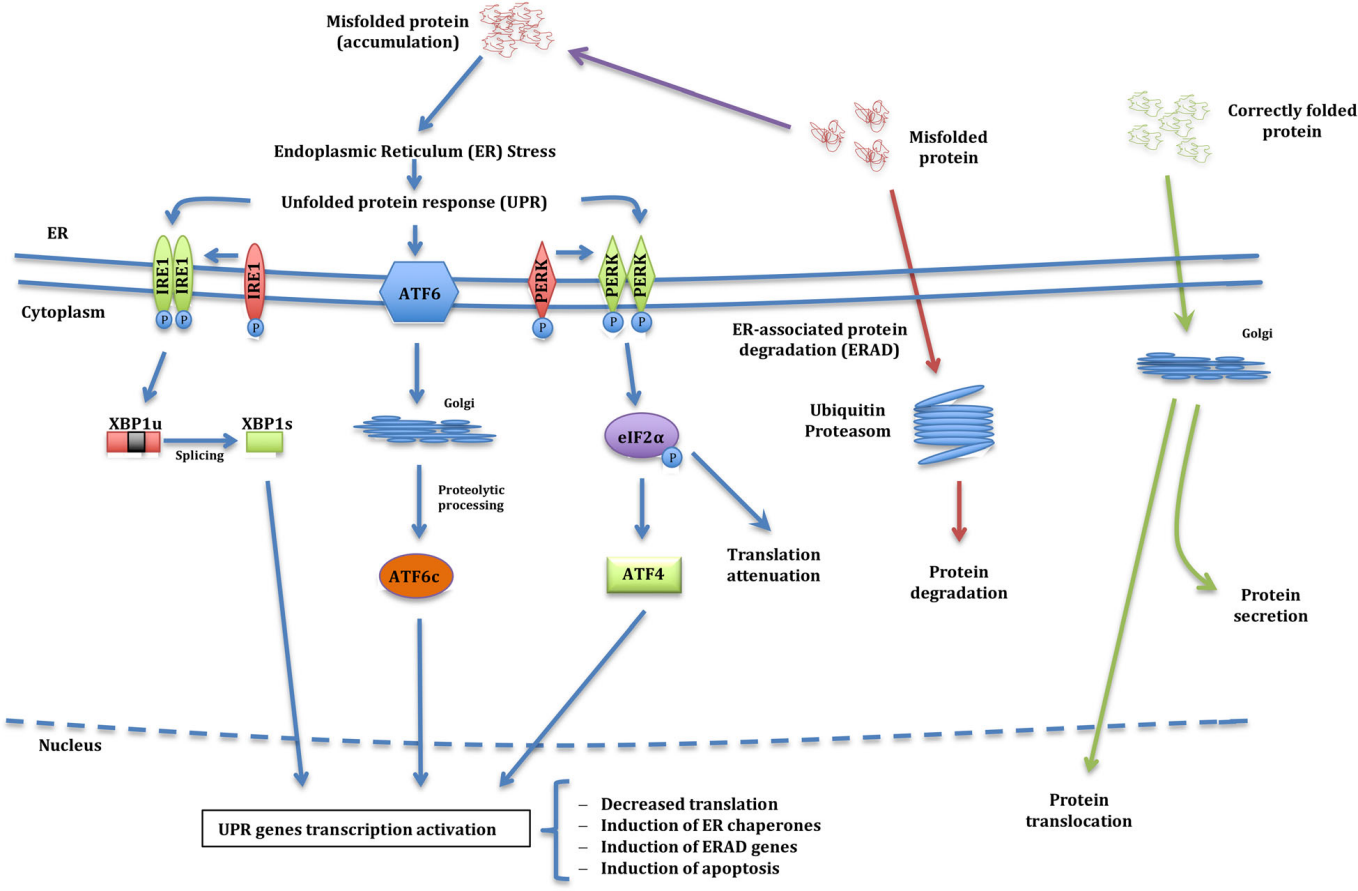
Endoplasmic reticulum (ER) stress and the unfolded protein response (UPR) initiation. Properly folded proteins (Green arrows) are processed at the Golgi apparatus and then translocated to their destination sites. Misfolded proteins (Red arrows) are retained in the ER lumen and are degraded by the ER-associated protein degradation machinery (ERAD). Under certain pathological situations misfolded proteins aggregate and accumulate into the ER lumen triggering a condition called ER stress (Blue arrows). In response to ER stress, the cell activates the Unfolded Protein Response (UPR), in which accumulated misfolded proteins are sensed by inositol-requiring enzyme 1 (IRE1), activating factor 6 (ATF6) and protein kinase R-like endoplasmic reticulum kinase (PERK) proteins. IRE1 protein dimerises, auto-phosphorylates and activates its endoribonuclease activity, which removes a small intron of the transcription factor X-box-binding protein 1 (XBP1u) that is then converted in XBP1s which acts as a transcriptional activator. ATF6 is cleaved and activated in the Golgi apparatus to yield a transcription factor (ATF6c) that migrates to the nucleus where activates the transcription of UPR target genes. PERK also dimerises and phosphorylates the eukaryotic translation initiation 2α (eIF2α), which attenuates most translation but stimulates translation of the transcription factor ATF4, which in turn activates genes to protect cells against the ER stress. The UPR signalling consists of four mechanisms: i) decreased translation to prevent further misfolded protein accumulation; ii) induction of ER chaperones to increase folding capacity; iii) induction of ERAD genes to increase degradation of misfolded proteins and iv) induction of apoptosis to remove stressed cells

Alpha-1 antitrypsin deficiency (AATD) is a rare hereditary condition characterised by low circulating levels of the alpha-1 antitrypsin (AAT) protein, a serine protease inhibitor synthesised and secreted mainly by hepatocytes, that protects lung tissues from damage caused by proteolytic enzymes such as neutrophil elastase (NE). The AAT protein is encoded by the *SERPINA1* gene and over 120 mutations have been reported at this locus [1, 2]. The commonest deficiency variants are the S and Z forms (as opposed to the normal wild-type M allele). The Z allele (both in homozygosis and heterozygosis) leads to misfolding and polymerisation of the protein, which accumulates in the endoplasmic reticulum (ER) of the hepatocytes, leading to chronic liver disease in some individuals. Hepatocyte damage is believed to be caused by ER stress, ER overload response, mitochondrial dysfunction and autophagy, although the pathophysiology is still unclear. Some AAT mutations (those that destabilise the protein dramatically) do not polymerise and cause ER stress, triggering the ER-associated protein degradation (ERAD) system and the unfolded protein response (UPR), (Fig. 1) whereas mutations that cause ordered polymerisation of the protein (such the Z allele) trigger an ER overload response that involves calcium-dependent nuclear factor (NF)-κB signalling and a pro-inflammatory response. The S mutated protein is retained within the hepatocytes although it does not form intrahepatic polymers unless the Z allele is present in keeping with less retention in the hepatocytes, absence of liver disease and intermediate plasma levels [3–5]. Although much of the misfolded protein is eliminated either by ERAD or by autophagy, a proportion is folded correctly and secreted into the circulation [6]. As a consequence, lower circulating plasma levels of AAT are found in patients with AATD, resulting in incapacity to inhibit NE efficiently. This leads to parenchymal lung destruction and chronic obstructive pulmonary disease (COPD) development, a situation that is exacerbated by smoking and occupational exposure to dust and fumes [1, 7, 8]. In rare cases, AATD has also been associated with other conditions such as necrotising panniculitis and systemic vasculitis (granulomatosis with polyangiitis; GPA) although this connection is less well established since a variety of genotypes, some with circulating values in the normal range, are associated with GPA [7, 9, 10].

Recent research has shown that AATD is characterised by neutrophilic inflammation and the disease is increasingly recognised as a neutrophil-driven inflammatory disorder both in the lung and with other systemic manifestations [11]. Beyond its antiprotease activity, AAT has anti-inflammatory and immunoregulatory features which open up a rationale for its potential use in other inflammatory conditions such as rheumatoid arthritis, diabetes mellitus, cystic fibrosis and asthma [12–14].

AATD is a highly underdiagnosed condition. Since the first symptoms resemble other respiratory pathologies, initial clinical diagnosis can be difficult especially in neonates and children [2]. A recent study has estimated the frequency of the PIS and PIZ alleles in 97 countries worldwide; more than 180,000 (0.1%) and 1.2 million (0.7%) individuals are estimated to have PIZZ and PISZ phenotypes respectively, most of them remaining undiagnosed [9, 15–17]. Early diagnosis is important to allow physicians to take preventive measures and initiate appropriate treatment when necessary [18]. Clinical data indicates that the severity of the symptoms found in AATD patients is highly variable and neither AAT serum levels nor phenotype are sufficient to identify which patients will develop severe lung or liver disease [19]. New strategies for early detection and biomarkers for patient outcome prediction are therefore needed to reduce morbidity and mortality in these patients.

Augmentation therapy is the only specific approved therapy to treat the pulmonary disease in patients with severe AATD [20]. However, the use of this therapy is controversial [21]. New treatment options are currently being investigated, including the use of gene therapy or induced pluripotent stem cells (IPSCs), and non-augmentation strategies to prevent AAT polymerisation inside hepatocytes.

In the light of the above, the AATD field is rapidly evolving with new and exciting discoveries. In order to summarise the current knowledge, identify areas of controversy and formulate questions that need further research a review of the scientific literature on AATD has been undertaken with a particular focus on recent advances in the field.

### Alpha-1 antitrypsin deficiency: A Paediatricians’ perspective

From a respiratory point of view, AATD is generally an adult-onset condition, so that there are usually no notable clinical differences between children with or without AATD. Recurrent respiratory manifestations in a child diagnosed with AATD are not necessarily caused by the disease, but may be an exacerbating factor in the progression of an underlying respiratory problem [22]. Therefore, paediatricians should aim to prevent respiratory infections and control the signs or symptoms of bronchial hyper-reactivity in these patients by administering the appropriate vaccines indicated for the child’s age, including hepatitis A and B, pneumococcal 13-valent vaccines, and an annual influenza vaccine.

Although AATD-associated liver disease can present from birth to old age, AATD is the most frequent cause of metabolic liver disease in paediatric patients [23–25] and the second most common indication for liver transplantation after biliary atresia [26]. The clinical course of AATD-related liver disease is highly variable and it is still unknown why some individuals develop AATD-related liver disease while others do not [27]. The majority of infants with homozygous severe AATD (PiZZ) are asymptomatic and clinically recover in early childhood; however, about 10–50% develop some form of liver abnormality, including elevated liver enzymes, cholestatic neonatal hepatitis, hepatomegaly and nutritional problems which may persist throughout childhood [28, 29]. Results from the Swedish newborn screening study have shown that the risk of life-threatening liver disease in childhood is approximately 5% [29]. In fact, only 2–3% develop fibrosis or cirrhosis requiring transplantation during childhood [30]. A recent systematic literature review was performed aimed at providing clarification on the clinical course of AATD in children and adults and to assess the clinical effectiveness of liver transplantation [27]. In children, liver cirrhosis was reported in 7.5% of patients, abnormal liver function tests in 9%, portal hypertension in 6.9%, jaundice in 1.9% and liver transplantation in 16.5%. No cases of hepatocellular carcinoma were reported, suggesting that it is a rare event. Risk factors for liver disease development such as serum bilirubin, pattern of clinical jaundice, portal hypertension and bile duct proliferation have been identified, but no clear pattern has been established. Mortality ranged from 0% in a small study of 10 PIZZ children who develop neonatal cholestasis and were followed until 20 years of age, to 25.5% in a cohort of 98 PIZZ/PISZ patients. Data also indicate that mortality due to AATD-associated liver disease has significantly decreased since the end of the 1980s, when liver transplantation became standard practice to treat patients with terminal illness associated with liver disease, and outcomes following liver transplantation were excellent regarding survival (74 to 92%) and quality of life in survivors, with no recurrence of liver disease or pulmonary complications, indicating that liver transplantation is an effective treatment for liver disease due to AATD [27].

These data, together with the fact that AATD is an autosomal codominant congenital disease, mean that paediatricians should aim to diagnose the disease in: i) all infants with persistent unconjugated hyperbilirubinemia, elevated transaminases, neonatal hepatitis syndrome, or other evidence of liver damage; ii) older children with chronic liver disease, cirrhosis, or portal hypertension; iii) children of patients with AATD [31].

### Diagnosis

#### Screening and laboratory and clinical diagnosis

Current recommendation documents and guidelines recommend/advise testing AAT levels in target populations, including individuals with COPD regardless of age or ethnicity, unexplained chronic liver disease, necrotising panniculitis, granulomatosis with polyangiitis, or unexplained bronchiectasis, and parents, siblings, and children, as well as the extended family of individuals identified with an abnormal gene for AAT. In these latter cases, AAT level testing alone is not recommended because it does not fully characterise disease risk from AATD although some guidelines advocate both AAT plasma levels and genotype for at least the S and Z alleles as initial testing [32–34].

Despite these recommendations AATD is a largely under-recognised condition [35]. Patients experience long diagnostic delays (up to 5.6 years) and often visit several doctors before the definitive diagnosis is reached [36]. With fewer than 10% of affected individuals being clinically diagnosed, AATD targeted detection is key to identifying potential cases [37]. Improving the use of this targeted detection starts by raising physician awareness [35]. Although typical cases tend to present at younger ages with lower lobe emphysema, in reality there is no single patient characteristic that can help raise suspicion: AATD cases have been detected in patients with different types of COPD, bronchiectasis, asthma, and in non-smoking individuals [38]. Newborn screening has several pros and cons and is currently not recommended, with the possible exception of countries with a high prevalence of AATD and smoking, where adequate counselling services are available [39]. In a nationwide neonatal screening for AATD carried out in Sweden between 1972 and 1974, 120 out of the 200,000 neonates screened were identified with a PIZ phenotype [28]. Follow up of this study has shown that the patients would rather know whether they are carrying a mutation since reduced smoking rates and cigarette smoke exposure in adulthood has been observed in patients that were diagnosed with AATD at birth [40, 41]. Based on these results and along with other considerations such as the high prevalence of the disease (1:6000–3500 similar to cystic fibrosis), the low cost of the diagnostic test, the diagnostic delay causing increased morbidity, and the existence of a treatment to delay lung disease progression, some authors consider that neonatal AATD diagnosis is appropriate [42]. On the other hand, other authors do not support neonatal screening reasoning that the financial and social costs outweigh the benefits and because there is no specific treatment for liver disease, which is the leading cause of childhood morbidity. Moreover, according to these authors, the reported changes in smoking behaviour in adulthood do not justify the social risks associated with neonatal AATD screening, such as family stress and inability to qualify for life insurance in some countries [43].

There is no single universally accepted laboratory algorithm for AATD diagnosis. According to current recommendations, quantitative serum AAT measurement in stable COPD patients is used as the initial screening test [38]. Recent publications have identified 104 mg/dL as a cut-off value for detecting PiZZ individuals with a negative predictive value of 99.8% [44]. However, there may be additional difficulties in identifying a threshold for detecting heterozygous carriers [45]. When the serum AAT concentration is lower than the reference range, the study should be completed with phenotyping and/or genotyping [46].

#### Stratification

Improved understanding of COPD pathogenesis together with new and better diagnostic techniques and increased clinicians’ awareness have shown that the clinical presentation of AATD-related COPD is not limited to purely emphysematous patients. Instead, as with non-AATD tobacco-related COPD, there is a wide range of disease presentations [47]. Accordingly, the confirmation of AATD should be followed by evaluating the specific clinical presentation in order to identify symptoms intensity and prognostic markers [48, 49].

Multidimensional tools and scales for determining AATD have been explored. The BODE (body mass index, airflow obstruction, dyspnoea, and exercise capacity) index was recently validated in a cohort of 191 AATD patients undergoing lung transplantation who were followed from 2006 to 2012. The authors found that the BODE index could better discriminate survival than both forced expiratory volume in one second (FEV_1_) alone and the 2011 Global Initiative for Chronic Obstructive Lung Disease (GOLD) classification. However, future trials will be required to elucidate the usefulness of the BODE index, or any other multidimensional scales, for treatment selection [50].

Additionally, different health status questionnaires and severity scores are available, including the St George’s Respiratory Questionnaire, the COPD severity score, the EuroQoL 5-Dimensions, the Living with COPD, and the COPD Assessment Test. Recently, an observational, cross-sectional study including 96 COPD patients (including 35 cases of AATD-related COPD) evaluated some of these questionnaires. Patients with AATD COPD showed a similar degree of health status impairment as those with non-AATD COPD. Additionally, there were stronger correlations between AATD COPD health status measurements and lung function impairment than for non-AATD COPD. Therefore, evidence regarding the performance of different questionnaires for more comprehensive evaluation of AATD patients is starting to accumulate [51].

### Prognosis

The natural history and prognosis of AATD is variable. Most people with a severe deficiency have a lower life expectancy relative to the general population [52, 53], with the exception of never-smokers that were identified through family or population screening [54]. The risk of developing AATD-related diseases depends not only on which AAT deficient alleles the individual carries, but also on other factors and modifiers including genetic polymorphisms that can modulate gene expression or environmental factors such as smoking, air pollution and dust exposure for lung disease or alcohol intake for hepatic injury.

Early diagnosis (and treatment) is the key to improving the prognosis of AATD-related disease [55], because it promotes smoking cessation [56] preventing young individuals developing a smoking habit and raising awareness to avoid exposure to occupational respiratory pollutants [57].

Respiratory disease is the main prognostic factor for most AATD patients and is predominantly represented by early-onset emphysema (58–72%) [52, 53]. Cigarette smoking has an adverse effect on the course of lung disease and is by far the single most important risk factor for the development of rapidly progressive COPD in patients with AATD [39, 58]. Epidemiological studies have shown that ever-smokers with severe AATD have increased emphysema, lower diffusing capacity of carbon monoxide (D_LCO_) values and increased airflow obstruction and sputum production than never-smokers [57, 59, 60]. Similarly, active smokers have a greater annual loss of lung function than never-smokers and ex-smokers [61, 62]. In a recent study it was shown that PISZ patients were less susceptible to cigarette smoke than PIZZ patients. Multivariate analysis revealed that PISZ patients were less likely to have emphysema and had better survival than PIZZ patients, given the same level of smoke exposure, although the lung function decline did not differ significantly [63].

The risk of lung disease in PIMZ individuals has been controversial for years. This is of particular importance because of the high prevalence of PIMZ individuals, meaning that even a moderate increase in the risk of COPD would have a significant public health impact. A meta-analysis showed an increased risk of COPD among PIMZ patients [64]. However, population-based studies showed no significant differences in FEV_1_ values between the PIMM and PIMZ groups, thus establishing an association between PIMZ and the development of COPD was complicated, partly due to the small number of patients included in these studies. However, later studies including a higher number of patients have demonstrated that ever-smokers PIMZ heterozygotes have and increased risk of COPD whereas there was no increased risk in never smokers. Moreover, in a family-based study it was shown that PIMZ individuals have a greater degree of airway obstruction than PIMM individuals with a similar degree of cigarette smoke exposure. Altogether, these results indicate that intensive counselling and PIMZ diagnosis is strongly advisable to avoid starting smoking in non-smokers or to help current smokers quit [56, 65, 66].

The severity of AATD-related liver disease is also highly variable. As noted above, it is the main clinical manifestation at paediatric ages but it can also affect adults, especially after the fifth decade of life, in some cases leading to severe forms of liver disease such as cirrhosis and hepatocellular carcinoma that may eventually require liver transplantation. Approximately 50% of PiZZ homozygotes show evidence of on-going inflammatory activity in the liver, and 2 to 43% develop cirrhosis [67]. The risk for adult liver disease increases with age. In a study analysing the age distribution of AATD as a cause of severe liver disease (as defined by the need for a liver transplant) the authors found that 77.2% of the patients were adults, with a peak age range of 50–64 years [68]. Several studies have shown that individuals with PIMZ phenotype have an increased risk of liver fibrosis or cirrhosis compared to the general population although it seems that alcohol consumption and non-alcoholic steatohepatitis are important factors in the development of liver disease in these patients [27].

Interestingly, adults with severe lung disease often do not develop liver disease and vice-versa. However, it has been shown that in adults, hepatic disease can coexist with pulmonary emphysema. In a study that included 57 patients with PiZZ AATD and established pulmonary disease, 63.2% had a history or clinical findings suggestive of liver disease and 17.5% showed evidence of advanced liver fibrosis [67].

### Augmentation therapy: Advances and controversies

Intravenous infusion of AAT in AATD individuals protects the lungs from the action of uncontrolled neutrophil elastase, and hence, slows the progression of emphysema [69]. However, although augmentation therapy has proved to have biochemical efficacy in reaching and maintaining protective AAT levels in blood and lung tissue, its clinical efficacy has been questioned [20]. Table 1 includes the most relevant studies analysing the clinical efficacy of AAT treatment.

**Table 1:** Studies on augmentation therapy

Authors	Dose	Type of study	End Point	Results
Non-randomised studies				
Seersholm et al., 1999 [58]	60 mg/kg/7 days	Observational, with control group (*n* = 295)	FEV1 decline	Less FEV1 decline in treated group (56 vs 75 ml/y; *p* = 0,02) Greater benefit for patients with FEV1:31–65%
American AAT Deficiency Registry Study Group, 1998 [50]	33%, weekly 43% biweekly 24% monthly	Observational, with control group (*n* = 1129)	FEV1 decline Mortality	Reduction of mortality (OR 0,64; p = 0,02) Less FEV1 decline in patients with FEV1 35–49%, treated (66 vs 93 mL/y; *p* = 0,03)
Wencker et al., 2001 [59]	60 mg/kg/7 days	Observational cohort. No control group (*n* = 96)	FEV1 decline	Less FEV1 decline during treatment (49,2 vs 34,2 mL/Y, p = 0,019). Lowest decline in FEV1 > 65% (256 vs 53 ml/Y, p = 0,001)
Tonelli et al., 2009 [60]		Observational with control group (*n* = 164)	FEV1 decline Mortality	Increase in FEV1: 10.6 ± 21.4 mL/Y; *p* = 0.05). No differences on mortality
Randomised studies				
Dirksen et al., 1999 [64]	250 mg/kg/28 days	RCT (*n* = 66) FEV1:30–80%	FEV1 decline, lung density	No significant effects on lung function Trend towards a favourable effect reducing loss of lung tissue
Dirksen et al., 2009 [68]	60 mg/kg/7 days	RCT (*n* = 77) FEV1:25–80%	Lung function, QoL, exacerbations, lung density	Reduction in loss of lung density measured by CT in treated patients (*p* = 0.049) No differences on FEV1 or DLCO No differences on exacerbations frequency
Chapman et al., 2015 [72]	60 mg/kg/7 days	RCT (*n* = 180) Pi*ZZ, rare or null genotypes AAT < 11 mM, Emphysema on CT, FEV1:35–70%	Lung function, QoL, exacerbations, lung density	Reduction in loss of lung density measured by CT in treated patients(p = 0,03). No differences on FEV1 or DLCO. No differences in QoL
Meta-analysis				
Chapman el al, 2009 [70]		Meta-analysis of studies on treated patients vs controls form Canadian Registry (*n* = 1509)	FEV1 decline	Reduction of 26% on FEV1 decline (17,9 ml/Y) in patients on treatment with ev AAT. Effect due to subjects with FEV1: 30–65%
Gotzsche and Johansen, 2010 [71]	60 mg/kg/7 days	Meta-analysis Cochrane from 2 RCT (*n* = 140)	FEV1 Decline DLCO Lung density Exacerbations	Lower lung density loss in treated patients (*p* = 0.03) No differences in lung function No differences in exacerbations
Stockley et al., 2010 [69]	60 mg/kg/7 days	Integrated analysis of lung density studies	Lung density loss FEV1 decline	Lower lung density loss in treated patients (1.73 vs 2.74 g/L, p = 0.006) No differences in FEV1 decline
Marciniuk et al., 2012 [63]		Meta-analysis of all studies including treated patients with ev AAT vs controls	All parameters	Reduction in lung density loss measured by CT. Reduction on mortality
Studies on exacerbations				
Lieberman, 2000 [73]	55% weekly 37% biweekly 8% monthly	Observational (online survey) *n* = 89	Exacerbations frequency	Reduction on exacerbations frequency from 3 to 5/year to 0–1/year on treatment with ev AAT
Stockley et al., 2002 [74]	60mgs/kg/7 days	Descriptive (*n* = 12)	Inflammatory biomarkers in sputum	Reduction of LTB4 after treatment
Barros-Tizón et al., 2012 [75]	180 mg/kg/21 days	Retrospective (pre-post AAT treatment)	Frequency and severity of exacerbations	Reduction on number and severity of exacerbations and hospital admissions related costs

Adapted from Casas F et al. Arch Bronconeumol 2015; 51:185–192 (ref. [38])

Early studies had FEV_1_ decline and mortality as the principal endpoint [62, 70–72] and they evidenced a reduction in FEV_1_ decline in the treated group. Larger observational studies showed that treatment with AAT augmentation therapy resulted in a slower decline in FEV_1_ and a reduction in mortality compared to those not receiving this treatment [70, 73, 74]. However, even though augmentation therapy was beneficial, the reduction in lung function loss was observed mainly for patients with a FEV_1_ between 35 and 60%, so this treatment was only recommended in patients that fall within this lung function-impairment range [39, 62]. Recently, other medical societies have proposed different criteria [38, 75].

One of the earlier randomised placebo-controlled trials studied the change in pulmonary function tests and lung density measured by CT, but only 30 patients were included and the study showed no difference in the pulmonary function tests. However, compared to the placebo group the change in lung density tended to improve (*p <* 0.07). The study showed that decline in FEV_1_ is not the appropriate method to assess the efficacy of augmentation therapy due to the large number of patients required [76]. Since then, the use of other markers such as D_LCO_ or lung density measured by computed tomography (CT) as alternative outcome metrics to FEV_1_ have been studied. More recent studies have reported that a decline in D_LCO_ is observed before FEV_1_ decreases [77], and that both D_LCO_ and lung density (as measured by CT) demonstrate lung parenchyma loss, even in severe disease where FEV_1_ may be stable [78]. Moreover, lung density assessed by CT also correlates with health-related quality of life (HRQL) and is the best predictor of mortality in AATD patients [79]. The EXACTLE randomised controlled trial [80] also evaluated changes in CT lung density in patients receiving AAT augmentation therapy versus placebo: the results were similar to the previous study and, although the differences were not significant, therapy also demonstrated a trend to improve lung density (*p* = 0.068). Data from these two clinical trials were pooled to increase the statistical power [81] showing a significant improvement in lung density decline (by 2.297 g/L in the treatment group) over two years in treated versus untreated patients (*p* = 0.006).

Whereas in some countries these data were sufficient for AAT augmentation treatment to become a registered treatment, others reject registration and reimbursement because of the lack of larger randomised, placebo-controlled trials. Indeed, despite several meta-analyses supporting the use of augmentation therapy [75, 81, 82] an unfavourable Cochrane review based on the rate of FEV_1_ decline [83] as well as the lack of consensus encouraged the search for new evidence. The RAPID trial gave additional information for the efficacy of augmentation therapy. This trial included 180 patients with emphysema secondary to AATD and a FEV_1_ of 35–70% (predicted), recruited in 28 centres in 13 countries [84]. The patients were randomised into augmentation therapy or placebo and followed for two years by CT densitometry. There was an additional extension in which all the patients received active treatment and were followed for an additional two years (RAPID-OLE) [85]**.** Primary endpoints in RAPID trial were CT lung density at total lung capacity (TLC) and at functional residual capacity (FRC) combined, and the two separately. Although the primary endpoint of lung density at TLC and FRC combined did not reach the statistical significance (*p* = 0.06), changes in CT lung density at TLC alone (another primary endpoint) showed a significant difference in the rate of lung parenchymal loss between patients who received augmentation therapy and those who received placebo (− 1.45 g/L per year versus − 2.19 g/L per year; *p* = 0.03), with an absolute difference of 0.75 g/L per year (95% CI: 0.06–1.42), corresponding to a relative reduction of 34% in favour of augmentation therapy. These results showed that augmentation therapy was effective in reducing annual lung tissue loss. Which was demonstrated by a statistically significant reduction of lung density loss measured at a total lung capacity (TLC) of 34% (*p* = 0.03). Moreover, patients who were initially in the placebo arm and agreed to participate in the extend study and subsequently received active treatment for the next two years, showed a reduction in their lung density decline rate similar to that of patients initially included in the active arm of the study [85].

Some studies have showed a reduction on exacerbation frequency and severity [86–88] in AATD patients under augmentation therapy (Table 1). However, some inconsistencies have been observed in the results obtained from these clinical trials indicating that further research is needed to clarify this point [31].

### Ongoing research and future treatments

#### Epigenetics and genetic modifiers

AATD symptoms and outcomes vary greatly, indicating that beyond the protease-antiprotease imbalance other genetic, epigenetic, and environmental and lifestyle factors may contribute to disease severity. Epigenetics refers to changes in gene expression not caused by DNA sequence changes. At the molecular level, three distinct but interconnected systems including DNA methylation, histone modification leading to chromatin remodelling, and non-coding RNAs are involved in epigenetic gene expression regulation. Understanding the mechanisms that are involved in the initiation, maintenance, and hereditability of the epigenetic changes observed in AATD is an important aspect of current research in this field [89].

DNA methylation is by far the best-studied form of epigenetic change. In one study, changes in the global DNA methylation pattern and systemic inflammation markers caused by cigarette smoke were analysed in 316 PiZZ AATD patients. The methylation levels of 16 CpG sites were significantly associated with an ever-smoking status, with all 16 being hypomethylated in this subset compared with never-smokers. However, after adjusting for age and sex, only one CpG site, in the transforming growth factor, β-induced (*TGFB1*) gene, was associated with ever-smoking. The same study found an association between C-reactive protein levels and changes in CpG sites in the runt-related transcription factor 3 (*RUNX3*), Janus kinase 3 (*JAK3*), and keratin-1 (*KRT1*) genes. Taken together, these results indicate that ever-smoking and age at smoking initiation is associated with both global and specific gene hypomethylation, and suggest that DNA methylation might be important in explaining disease heterogeneity [90]. Similarly, DNA methylation was associated with both the presence and severity of COPD in two family-based cohorts comprising 1.085 and 369 subjects, respectively. Although none of the subjects included in the studies were PIZZ a hypomethylation of the SERPINA1 gene at loci cg02181506 was associated with COPD and with poor lung-function phenotypes [91]. Additionally, the methylation patterns and AAT gene expression were studied in two series of somatic cell hybrids between a rat hepatoma line and human fetal liver fibroblasts or human skin fibroblasts. The results indicate a clear correlation of hypomethylation with increased AAT gene expression, while inactive AAT genes were highly methylated. Nevertheless, the functional meaning of this change is currently unknown in humans [92]. Altogether, these studies show a link between changes in the DNA methylation pattern and phenotype and severity of AATD.

MicroRNAs (miRNAs) are short non-coding, single-stranded RNA molecules which act at the post-transcriptional level and play key roles in regulating gene expression. So far, the role of miRNAs in AATD has been very little studied. miRNA expression and function were analysed in monocytes isolated from both symptomatic and asymptomatic PiMM and PiZZ individuals. The authors described a group of 43 differentially expressed miRNAs and showed that miR-199a-5p may be an important regulator of both unfolded protein response and inflammation in AATD. These investigators showed that miR-199a-5p is the most up-regulated miRNA in asymptomatic PiZZ vs. PiMM monocytes, but conversely, miR-119a-5p expression was decreased in symptomatic PiZZ patients, a process mediated by hypermethylation of the miR-119a-2 promoter [93, 94]. In a recent study, gene and miRNA expression were analysed in PBMCs of a small group of PIZZ-AATD patients with severe (*n* = 6) and mild (n = 6) COPD. The authors identified that patients with severe COPD–AATD disease presented 205 differentially expressed mRNAs (114 upregulated and 91 downregulated) and 28 miRNAs (20 upregulated and 8 downregulated) compared to patients with mild disease. Of these down-regulated miRNAs in severe emphysema patients, miR-486 and miR-335 have previously been related to respiratory diseases. Downregulation of miR-335 involves the activation of pathways related to inflammation and angiogenesis. Therefore, these results suggest a correlation between decreased miR-335 expression and severity of AATD-related emphysema. However, this finding must be confirmed in large studies including a control group of patients with non- AATD–related COPD. [95]. Overall, these studies provide additional information on the role of miRNA in AATD, which is related to the development and progression of the disease.

As previously mentioned, AATD is caused by mutations in the AAT gene leading to protein misfolding. Proper protein folding is carried out by a complex network of proteins and pathways called the proteostasis network, a process regulated by several signalling pathways including the oxidative stress (OS) and inflammatory signalling pathways and the acetylation proteostasis system. Histone acetyltransferase and deacetylases (HDACs) have been shown to play important roles in liver and lung physiology by modifying the acetylation–deacetylation equilibrium, including in AATD. One report described correcting the Z form of AAT secretion in response to treatment with the HDAC inhibitor suberoylanilide hydroxamic acid (SAHA) which restored Z-AAT secretion and serpin activity to 50% of wild-type AAT levels, thus suggesting that SAHA may be a potential treatment for AATD [96].

Several studies have shown that OS may be involved in the pathogenesis of AATD. Recent studies by our research group have shown that OS produced by a reduction of antioxidant defences is involved in the pathophysiology of AATD at early ages, before relevant clinical manifestations have occurred, and is associated with a higher risk of developing lung and/or liver disease [97]. Further studies demonstrated that increased OS leads to telomere attrition in AATD patients and an association between telomere length and AAT phenotypes, suggesting that telomere length could be a promising biomarker for AATD disease progression [98]. In a mouse model, exposure to cigarette smoke accelerates polymerisation of Z-AAT by oxidative modification of the AAT protein and enhances the neutrophil influx into the lungs [99]. Another study using Hepa1.6 cells has shown that disulphide interactions enhance intracellular accumulation of AAT, while treatment of cells with reducing agents increase Z-AAT secretion [100]. Altogether, these studies link redox states with polymerisation and intracellular retention of AAT, suggesting that redox state is a modifier factor for AATD and that targeting OS may be a promising therapeutic option for these patients [101, 102].

Single nucleotide polymorphisms (SNPs) in the endothelial nitric oxide synthase (NOS3) [103], glutathione s-transferase p1 (GSTP1) [104, 105], tumour necrosis factor alpha (TNFA) [106], interleukin 10 (IL10) [107], microsomal epoxy hydrolase (mEH) [105], cholinergic nicotine receptor alpha3 (CHRNA3), and iron regulatory binding protein 2 (IREB2) [108] genes have all been shown to influence COPD development in AATD patients [108].

#### Biomarkers

Biomarkers which can act as an indicator of normal lung or liver physiology, disease progression, or response to AAT augmentation therapy, are being evaluated in the AATD field [109]. Serum gamma glutamyl transferase (GGT) is used in clinical practice as a marker of liver disease. It is transiently elevated in PIZ children although it is a bad predictor of future liver problems in AATD patients [97, 98, 110]. Recent research has shown that serum GGT is independently associated to the severity of lung disease and respiratory mortality suggesting that might be a novel marker for respiratory disease in AATD patients [111].

Desmosine and isodesmosine are well-studied lung elastin degradation biomarkers which appear alongside the development of COPD. Preliminary studies showed that desmosine and isodesmosine levels in biofluids (plasma, urine, and sputum) from COPD patients with or without AATD are increased [112, 113]; one study also showed evidence that AAT augmentation therapy diminished desmosine excretion in AATD patients [114].

Circulating polymers can be used to diagnose AATD and are being investigated as prognostic biomarkers of the disease. Current data indicate that they may be involved in lung function decline in AATD patients. However, further studies to establish the stability of circulating polymers and its value as prognostic biomarkers are needed [115].

Fibrinogen has been recognised as a COPD biomarker [116]. Fibrinogen levels are related to the presence and frequency of exacerbations, disease severity and mortality in COPD patients [117]. Similarly, a specific blood fibrinogen (Aa-Val360) degradation product is increased in AATD patients, indicating airflow obstruction severity, and decreases in subjects receiving AAT augmentation therapy [118]. Results so far indicate that it may be a useful disease activity marker in patients with early disease in whom therapeutic intervention may be indicated [119].

Beyond their role as regulatory molecules, miRNAs are also being investigated as disease biomarkers in several lung [120] and liver pathologies [121]. In a preliminary study, plasma miRNA profile analysis in AATD individuals revealed a genetic signature that discriminate between the different AATD risk groups [122].

#### Emerging therapeutic strategies

AAT augmentation therapy requires regular intravenous infusion of plasma-purified AAT, which is costly and dependent on the availability of the protein. Therefore, alternative strategies are currently being investigated, including new delivery strategies, the use of gene therapy or iPSCs, non-augmentation strategies to prevent AAT polymerisation inside hepatocytes, the use of autophagy-enhancing drugs and silencing RNA strategies [123, 124].

Aerosol delivery is being investigated as an alternative, more effective method to deliver AAT to the lung. Early studies in humans have shown biochemical efficacy and safety, although larger clinical trials are needed [125].

**Fig. 2:**
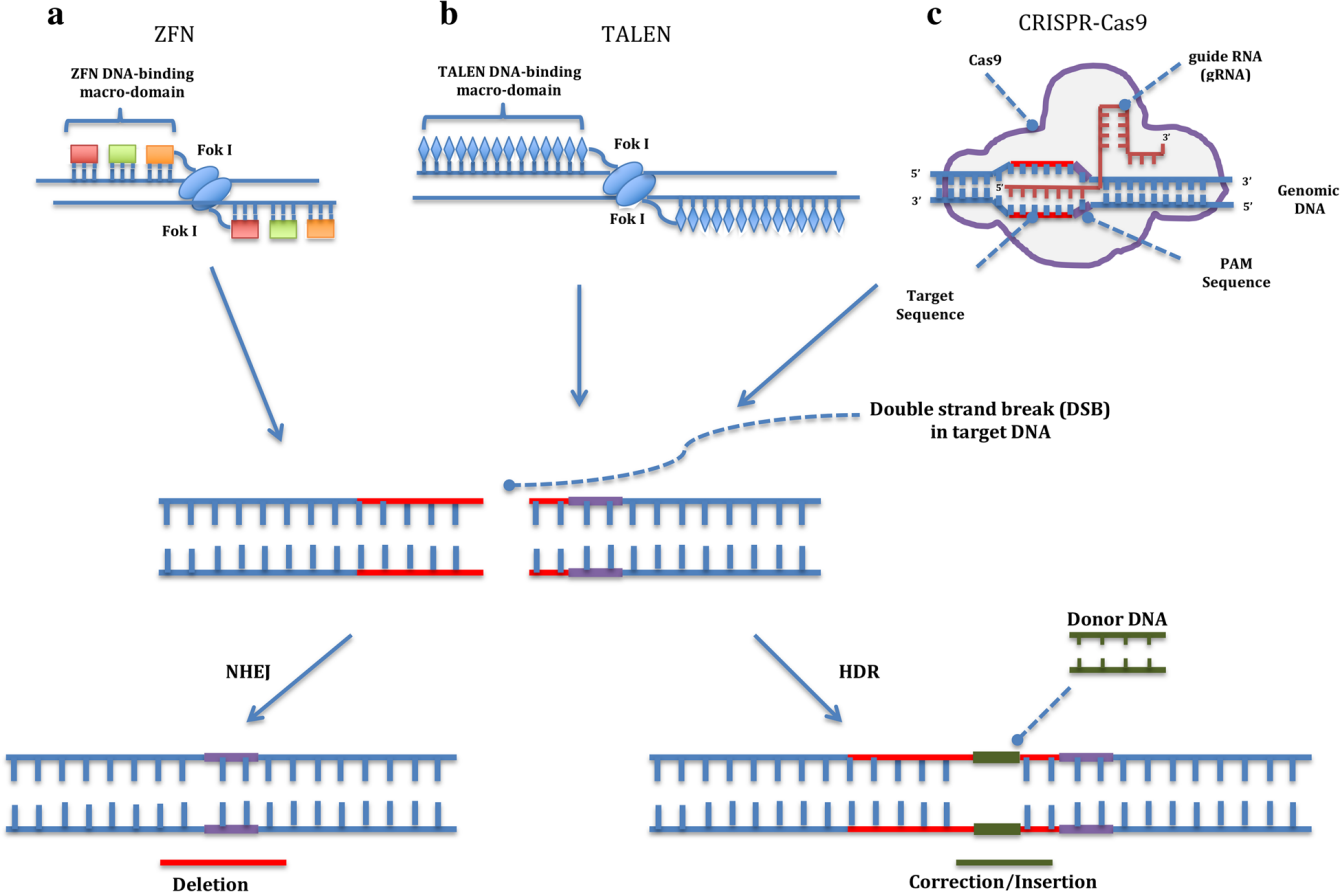
Genome editing with engineered nucleases. Genome editing involves two steps: i) a nuclease is engineered to cleave a specific (target) sequence in the DNA creating a double strand break (DSB); ii) the cell’s ability to repair the DSB by non-homologous end-joining (NHEJ) causes a deletion in the target gene that can result in gene mutation or complete knockout whereas homology-directed repair (HDR) by homologous recombination using a homologous DNA template results in gene correction or insertion depending on the DNA donor structure. There are three main classes of engineered nucleases. **a** Zinc finger nucleases (ZFNs) consist of a DNA-binding macro-domain designed to target the sequence of interest that is composed of several zinc-fingers each one recognising three nucleotides in the target sequence and linked to the nuclease domain of the FokI restriction enzyme. After dimerisation of two ZFNs in inverse orientation and with an optimal spacing of 5–7 nucleotides, the dimeric FokI cleaves the DNA between the binding sites. **b** Transcription activator-like effector nucleases (TALENs) have a similar structure to that of ZFNs. The TALEN DNA-binding macro-domain is composed of a tandem array of 34 aminoacids each recognising a single nucleotide. Similarly to ZFNs, TALENs also depend on FoKI activity and dimerisation to create a DSB between the binding sites. **c** In the CRISPR-Cas9 system, a site-specific DNA cleavage is performed by nuclease Cas9 directed by complementary between an engineered single guide RNA (gRNA) and the target sequence

**Fig. 3:**
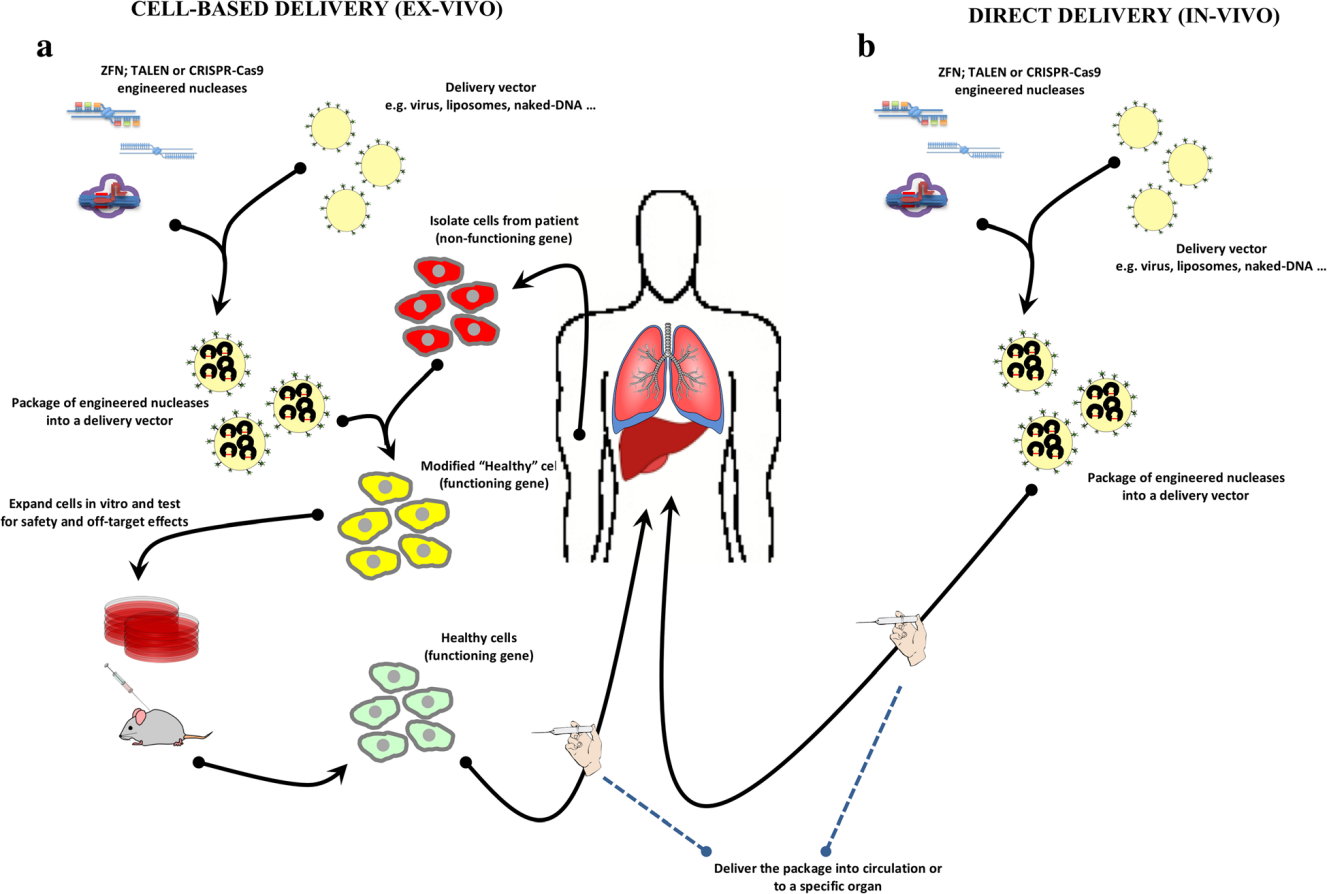
Strategies for delivery of engineered nucleases. **a** Cell-based (ex-vivo) approach. The therapeutic engineered nucleases are packaged into a delivery vehicle (virus, liposomes, naked-DNA, etc). Cells from patient carrying the mutated non-functioning gene are isolated and transfected with engineered nucleases to correct the mutated gene. Modified “healthy” cells are expanded in vitro and test for safety and off-target effects before being re-administered to the patient. **b** Direct-delivery (in vivo) approach. In that case, the therapeutic engineered nucleases are packaged into a delivery vehicle (virus, liposomes, naked-DNA, etc) and injected directly into the patient

Replacement strategies using gene therapy in animal models using viral [126] and non-viral gene [127, 128] transfer methods were first reported years ago, but this strategy would only be useful for treating emphysema because it cannot be used to treat liver disease. However, two recent studies using transgenic mouse AATD models have shown that Z gene expression can be knocked out while inserting the gene encoding wild type (WT) AAT. High therapeutic levels of human AAT and a simultaneous and significant reduction in the hepatic accumulation of Z protein were observed, although the reduction was not sufficient to prevent liver fibrosis [129, 130]. The recent advent of efficient genome editing based on zinc-finger nucleases, TALENs and the CRISPR/Cas9 system has opened up new strategies for definitive gene correction of the Z-AAT mutation in hepatocytes, which are currently under investigation. These techniques are based on chimeric endonucleases targeted to a specific site within the genome, where a double strand break (DSB) is provoked. The DSB can be repaired either by non-homologous end-joining (NHEJ) or by homology directed repair (HDR) mechanisms. In the NHEJ pathway, the break ends are ligated without the need for a homologous DNA donor template leading most of the times to gene inactivation. In contrast, HDR is based on homologous recombination mechanisms and requires a foreign DNA donor template with sufficient homology to the genome on both sides of the region to be modified to guide gene edition. These homologous sequences can recombine into the chromosome, replacing the endogenous sequence with the new DNA so that the desired genomic alteration (replacement, insertion or deletion) can be achieved. This way, small insertions or deletions -if NHEJ occurs- or specific changes -if HDR occurs- can be introduced on the genomic sequence of interest (Figs. 2 & 3) [131, 132]. However, before these techniques can be used in clinical settings some key questions must be resolved. Some aspects, such as targeted delivery to hepatocytes and optimisation of gene editing efficiencies to achieve physiological effects, need further investigation. Another important aspect to be resolved is the prevention of the recent reported off-targeted mutagenesis [133]. However, new methods to improve gene editing specificity are under study and have already yielded promising results [134, 135].

An alternative approach is to take advantage of the higher proliferative capacity of WT-AAT hepatocytes over their PiZZ counterparts; using a PiZZ mouse model, Ding et al. demonstrated that WT hepatocytes can be transplanted to the diseased liver where they then substitute PiZZ hepatocytes [136]. Building on this finding, the AAT gene Z mutation has been corrected in hepatocyte-like cells derived from iPSCs, and these cells were then transplanted into a mouse liver to produce sustained levels of human AAT in vivo. However, this type of therapy also carries the risk of introducing potentially harmful point mutations, and the accumulation of epigenetic changes in these cells cannot be excluded, which for now precludes the use of this technique in clinical practice at this stage of its development [137, 138].

Several strategies to prevent the polymerisation of mutated forms are also currently being studied. One peptide that targets a lateral hydrophobic area of the mutated AAT-Z protein was found to prevent polymerisation, although it increased intracellular degradation of the protein rather than inhibiting its secretion [123, 139, 140]. Similarly, reactive loop analogue peptides increase the secretion rate of the mutated forms but seem to increase their intracellular accumulation [140, 141].

Autophagy enhancement as a therapeutic alternative to liver transplantation has attracted a lot of interest recently. The autophagy-enhancing drugs carbamazepine and rapamycin stimulate intracellular degradation of misfolded Z-AAT and decrease hepatic fibrosis in a mouse model of AATD-associated liver disease [142, 143]. Carbamazepine is currently being tested in phase 2/3 pilot, in a double-blind, placebo-controlled, randomised, clinical trial for severe liver disease attributable to AATD [144].

Another non-augmentation strategy involves the use of interference RNA (RNAi) to silence Z-AAT in hepatocytes. Preclinical data indicates that chronic silencing reduces inclusion body formation and hepatic damage in a mouse model of the disease [123].

## Conclusions

In summary, AATD remains underdiagnosed. Therefore, new strategies to enhance detection are needed, especially because available evidence supports the clinical efficacy of augmentation therapy and promising new alternative therapies are currently being investigated that may change the panorama of treatment and disease over the next few years. In addition, relevant biomarkers are still needed to stratify patients to better predict disease progression rates or monitor response to treatment. The clinical utility of these biomarkers will increase as our understanding of the molecular mechanisms involved in emphysema moves forward.

## Abbreviations

AAT: Alpha-1 antitrypsin
AATD: Alpha-1 antitrypsin deficiency
ATS: American Thoracic Society
COPD: Chronic Obstructive Pulmonary Disease
CT: Computed Tomography
DLCO: Diffusing Capacity of Carbon Monoxide
ERS: European Respiratory Society
FEV1: Flow Expiratory Volume in 1 s
GGT: Gamma Glutamyl Transferase
HDACs: Histone acetyltransferase and deacetylases
IPSCs: Induced Pluripotent Stem Cells
NE: Neutrophil elastase
SAHA: Suberoylanilide hydroxamic acid
WHO: World Health Organization
